# PD‐0332991 combined with cisplatin inhibits nonsmall cell lung cancer and reversal of cisplatin resistance

**DOI:** 10.1111/1759-7714.13866

**Published:** 2021-02-03

**Authors:** Minghui Liu, Liyuan Cui, Xin Li, Chunqiu Xia, Yongwen Li, Rui Wang, Fan Ren, Hongyu Liu, Jun Chen

**Affiliations:** ^1^ Department of Lung Cancer Surgery, Tianjin Key Laboratory of Lung Cancer Metastasis and Tumor Microenvironment, Tianjin Lung Cancer Institute Tianjin Medical University General Hospital Tianjin China; ^2^ Emergency Department Tianjin Medical University General Hospital Tianjin China; ^3^ Department of Thoracic Surgery, First Affiliated Hospital, School of Medicine Shihezi University Shihezi China

**Keywords:** chemotherapy, cisplatin, drug resistance, lung cancer, PD‐0332991

## Abstract

**Background:** Acquired resistance of chemotherapy, especially cisplatin, is a major challenge in lung cancer treatment. We conducted this study to examine whether a cyclin D kinase 4/6 (CDK4/6) inhibitor, PD 0332991, could reverse cisplatin resistance in human lung cancer cells. In addition, we explored the underlying mechanisms.

**Method:** We used CCK‐8 assay to got the IC50 of PD‐0332991 and cisplatin in A549 and A549/CDDP respectively. CCK‐8 assay, CalcuSyn 2.0 software, cell cycle distribution and apoptosis used to identify PD‐0332991 could reverse the acquired resistance of cisplatin. At last, western‐blot used to show the mechanism of PD‐0332991 enhances the effects of cisplatin.

**Results:** We found that PD‐0332991 potentiated cisplatin‐induced growth inhibition in both cisplatin‐sensitive (A549) and cisplatin‐resistant (A549/CDDP) cells via downregulation of the proliferation, induction of apoptosis (A549 increased to 7.06%; A549/CDDP increased to 7.03%), and G0/G1 cell cycle arrest (A549 increased to 9.15%; A549/CDDP increased to 49.92%). Western blot analysis revealed that PD‐0332991 enhance the effect of cisplatin through inhibit Rb‐E2Fs pathway.

**Conclusions:** These findings suggest that PD‐0332991 could reverse the acquired resistance of cisplatin in lung cancer cells and provide a novel treatment strategy for lung cancer patients with cisplatin resistance.

## Graphical abstract

Acquired resistance of cisplatin is associated with dysregulation of cell cycle. CDK4/6 inhibitor, PD‐0332991, could reverse the resistance of cisplatin.

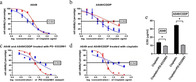

## INTRODUCTION

Among all types of cancer, lung cancer has the highest morbidity and mortality rates in China. As per recent reports, the number of lung cancer patients in China has exceeded 700 000 and the number of deaths has reached 600 000 annually.^1^ Nonsmall cell lung cancer (NSCLC) accounts for >80% of lung cancer cases.^2^ Most patients are diagnosed with advanced lung cancer and are not eligible for surgery. Thus, platinum‐based chemotherapy plays a significant role in the treatment of advanced lung cancer. The 5‐year survival rate of lung cancer patients remains relatively low (<10%).^3^ The reason is easier to develop drug resistance and most treatment strategy are less contribute to improve 5‐year survival rate of lung cancer.^4^, ^5^, ^6^, ^7^ It is therefore important to identify the detailed mechanism of cisplatin resistance, reverse the resistance, and improve the efficacy rate in the treatment of NSCLC.

Our previous research revealed that when an anticancer drug is administered, a small proportion of the cells survives, escapes from the cell cycle, and enters into a quiescent stage (G0). In certain situations, the quiescent cancer cells return to the cell cycle from the G0 phase. This is termed the “cell cycle re‐entry” theory; we successfully applied this theory in epidermal growth factor receptor‐tyrosine kinase inhibitors (EGFR‐TKI)‐acquired resistance.^8^, ^9^, ^10^ In this model, cisplatin could kill most lung cancer cells; however, the remaining cells are forced into the G0 phase and escape from cisplatin damage. When the microenvironment changes, the cancer cells may re‐enter the cell cycle and start proliferation.

While as the theory we elucidate above about “cell cycle re‐entry” theory, we also thought targeting the cell cycle could reverse cisplatin resistance as done previously in EGFR‐TKI. Thus, we used palbociclib (PD‐0332991),^11^, ^12^ which is an orally active small molecule that potently and specifically inhibits cyclin D kinase 4/6 (CDK4/6) in a reversible manner, working to reverse cisplatin resistance.

## MATERIALS AND METHODS

### Materials

Human lung adenocarcinoma A549 cell line (American Tissue Culture Collection, ATCC); human lung adenocarcinoma A549/CDDP (Tianjin Lung Cancer Institute); RPMI‐1640 Medium, FBS, and DMSO (Gibco); AnnexinV‐FITC/PI (KGI Bio); BCA Protein Assay Kit and Cell Counting Kit‐8 (Biyuntian Biotechnology); TEMED, glycine, Tris–HCl, SDS, and Tween‐20 (Beijing Dingguo Changsheng Bio); flow cytometer (American BD); full‐wavelength microplate reader (Molecular Devices); Western blot instrument (German Bole Company); cisplatin (Shandong Qilu Pharmaceutical); PD‐0332991 (American ApexBio); CDK4, CDK6, cyclin D1, Rb, Phospho‐Rb, Bcl‐2, E2F1, and caspase‐3 antibody (Cell Signaling Technology); protein membrane (PALL Corporation); cell culture dish and 6‐well and 96‐well plates (Greiner Bio‐One); and EP tube, centrifuge tube, and pipette (Axygen).

### Method

#### Cell culture

A549 (human lung adenocarcinoma cell line obtained from the ATCC) and A549/CDDP (cisplatin resistance cell line incubated by our institute) cell lines were cultured in an RPMI‐1640 medium containing 10% fetal bovine serum and incubated at 37 °C at 5% CO_2_ saturated humidity in the A549/CDDP cell culture medium. The drug resistance of the A549/CDDP cells was maintained by adding 1 mg/ml of cisplatin. When the density of cells reached 80%, the cells were digested with trypsin and selected in the logarithmic growth phase for subsequent experiments.

#### CCK‐8 cell activity assay

A549 and A549/CDDP cell proliferation was determined using a Cell Counting Kit‐8 (CCK‐8, Beyotime Institute of Biotechnology) according to the manufacturer's instructions. For the CCK‐8 assay, 2 × 104 cells/well were seeded in a 96‐well plate for 12 h and added drugs in different concentrations (0, 1, 2, 4, 8, 16, 32, 64, and 128 mg/ml) and PD‐0332991 (0, 1, 2.5, 5, 10, 20, 40, 80, and 160 mmol/L). After 24 h, we added the medium containing 10% CCK‐8 into each well. Then the 96‐well plates were incubated for 2 h at 37 °C, and the absorbencies of each group were measured at 450 nm by a microplate reader. All experiments were biologically repeated at least three times. The obtained results were calculated using the GraphPad Prism 5.0 software for the IC50 of each group.

#### Flow cytometry analysis of the cell cycle

To determine the effects of cisplatin and/or PD‐0332991 on the cell cycle, 1.5 × 105 cells/well were seeded in 6‐well plates and incubated for 12 h. Cells were synchronized by starving them in serum‐free RPMI‐1640 medium for 24 h. Cells were then incubated with or without cisplatin and/or PD‐ 0332991 for 24 h each, trypsinized, and fixed in 70% ice‐cold ethanol overnight. Cells were then treated with DNase‐free ribonuclease (Takara), stained with propidium iodide (PI) (Sigma–Aldrich), and analyzed using a FACSAriaTM flow cytometer (BD Bioscience) and ModFit LT software (Topsham).

#### Flow cytometry analysis of apoptotic cells

Cells (2 × 105 cells/well) were seeded into 6‐well plates and cultured for 12 h. Cisplatin and/or PD‐0332991 were added at various concentrations and cells were cultured for another 24 h. Cells were stained using an Annexin V‐FITC Apoptosis Analysis Kit (BD Bioscience) and analyzed with a FACSAriaTM flow cytometer (BD Bioscience).

#### Western blot analysis

A549 and A549/CDDP cells were treated with 1/2 IC50 of cisplatin and/or PD‐0332991 for 24 h. Then cells were homogenized in RIPA buffer (50 mM Tris–HCl, pH 7.4, 150 mM NaCl, 1% Nonidet P‐40, 0.5% sodium deoxycholate, 0.1% SDS, 1 mM EDTA, 1 mM PMSF, 1 mg/ml Aprotinin), and proteins quantified using the bicinchoninic acid (BCA) protein assay kit (Pierce). A total of 10–50 μg of proteins was fractionated on 10–12% gels using SDS‐PAGE, transferred to nitrocellulose membranes (Amersham Biosciences) under wet conditions, then immunoblotted with the appropriate antibodies.

#### Statistical analysis

All data were analyzed by using the Statistical Package for Social Sciences (version 15.0.1, SPSS Inc.). Student's *t*‐tests were used to identify the statistical significance between groups. ANOVAs were used for analysis of more than two groups of data in all experiments. Statistical significance: *p* < 0.05.

## RESULTS

### The combined effects of PD‐0332991 and cisplatin on the A549 and A549/CDDP cells

**FIGURE 1:**
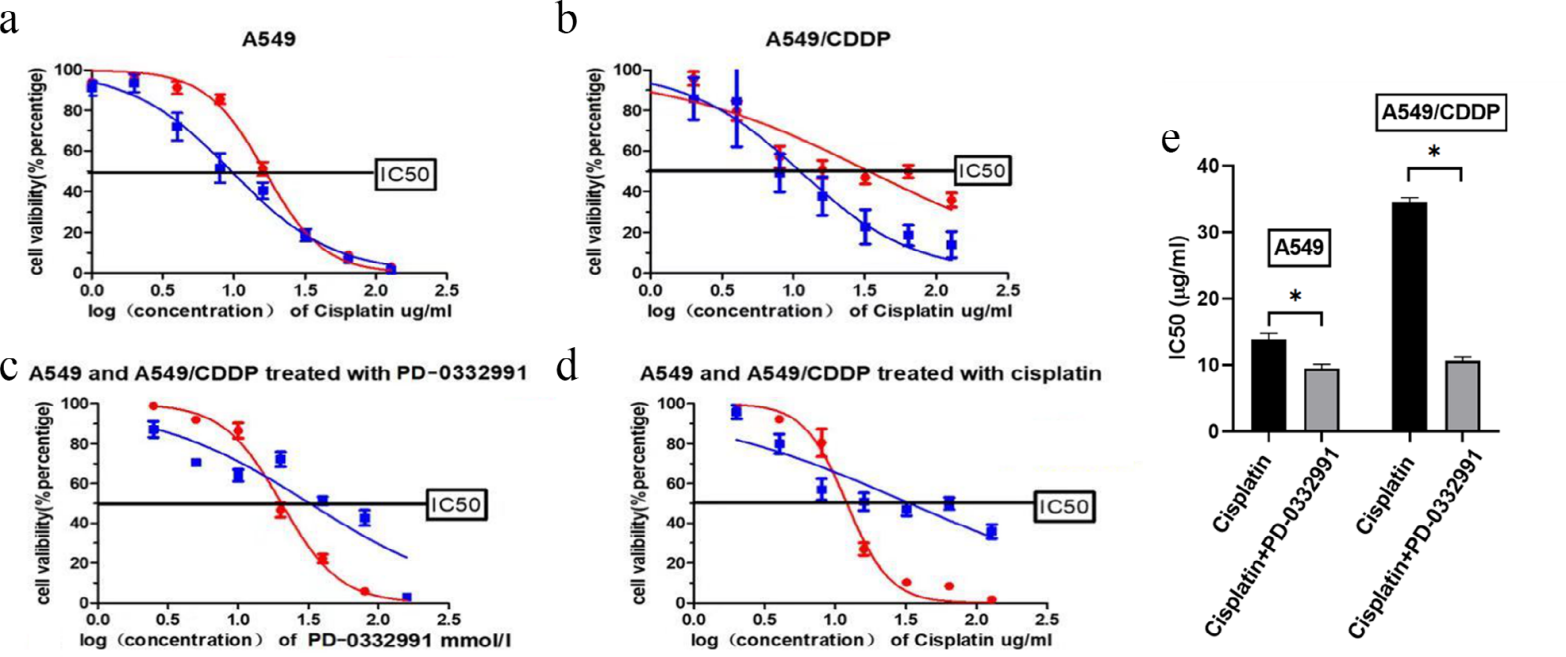
PD‐0332991 enhances the growth inhibitory effects of cisplatin in A549 and A549/CDDP cells. (a) The A549 and A549/CDDP cells were treated with different concentrations of PD‐0332991 (0, 2.5, 5, 10, 20, 40, 80, and 160 μmol/L) for 24 h and then assayed for cell viability. (b) The A549 and A549/CDDP cells were treated with different concentrations of cisplatin (0, 1, 2, 4, 8, 16, 32, 64, 128, and 256 μg/ml) for 24 h, then the cell viability curve was determined using CCK‐8. (c) The A549 cells were treated with different concentrations of cisplatin and half dosage of IC50 concentration of PD‐0332991 for 24 h. (d) The A549/CDDP cells were treated with different concentrations of cisplatin and different concentrations of cisplatin and half dosage of IC50 concentration of PD‐0332991 for 24 h. (e) The IC50 values of the cells treated with the combination of cisplatin and PD‐0332991 were lower than those of the cells treated with cisplatin alone

First, we used CCK‐8 to obtain the IC50 of cisplatin and PD‐0332991 in the A549 and A549/CDDP cells (Figure 1(a),(b)). After exposure to cisplatin for 24 H, A549 cells remained sensitive to treatment with an IC50 of 16.48 μmol/L, while PC‐9/AB2 cells were less sensitive with an IC50 of 33.85 μmol/L. The IC50 values of the A549 and A549/CDDP cells after treatment with PD‐0332991 for 24 h were 23.08 and 30.7 μmol/L, respectively.

To observe the synergistic effect of PD‐0332991 and cisplatin, the A549 and A549/CDDP cells were treated with 11.5 umol/L and 15.35 umol/L respectively. Cell viability was detected using CCK8. We found that the inhibitory effect on lung cancer cells was significantly enhanced when the two drugs were combined (Figure 1(c),(d)), and the IC50 value of cisplatin + PD‐0332991 was lower than those of A549 and A549/CDDP, irrespective of the cell type (Figure 1(e)).

### Synergistic effect of PD‐0332991 and cisplatin

**FIGURE 2:**
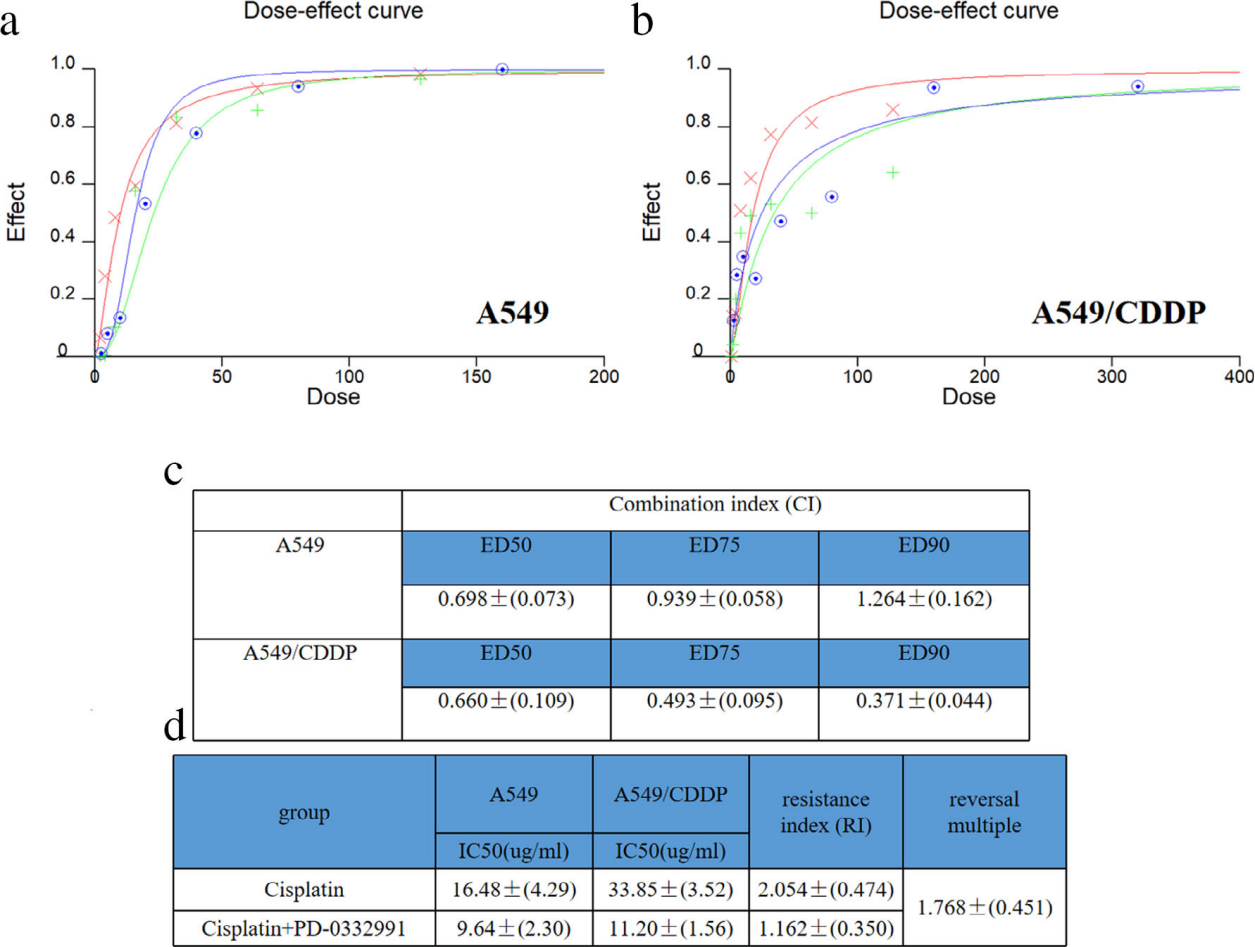
The synergistic effect of PD‐0332991 and cisplatin was statistically proven. (a) Combination of cisplatin and PD‐0332991 in the A549 cells. (b) Combination of cisplatin and PD‐0332991 in the A549/CDDP cells. (c) Combination index (CI) of cisplatin and PD‐0332991 at ED50, ED75, and ED90. (CI = 1, the two drugs have additive effects; CI < 1, the two drugs have synergistic effect; and CI > 1, the two drugs have a repulsive effect). (d) The IC50 values of the two cells with or without PD‐0332991, and the resistance index (RI) and drug resistance reversal multiple. RI = IC50A549CDDP/IC50A549

To further prove the synergistic effect of PD‐0332991 and cisplatin on the A549 and A549/CDDP cells, we used CalcuSyn 2.0 software. The results revealed that low doses of PD‐0332991 and cisplatin exerted a more synergistic effect compared with high doses on the A549 cell line (Figure 2(a)). In contrast, in the A549/CDDP cell line, the synergistic effect and drug dose were positively correlated (Figure 2(b)). We used the combination index (CI) at ED50, ED75, and ED90 to investigate the combination effect of cisplatin and PD‐0332991 (Figure 2(c)). In the A549 cells, the 50%, 75%, and 90% effect doses were 0.69837, 0.93901, and 1.26464, respectively. At ED90, cisplatin and PD‐0332991 exhibited no synergistic effect. However, in the A549/CDDP cells, the 50%, 75%, and 90% effect doses were 0.66038, 0.49314, and 0.37029, respectively. As the effect dose increases, the CI decreases. So neither in A549 or in A549/CDDP, PD‐0332991 combined with cisplatin could exert a synergistic effect. The resistance index (RI) of the A549/CDDP cells to cisplatin was obtained. The drug RI was 2.054 in the cisplatin group and 1.162 in the combination group, indicating that PD‐0332991 significantly reduced the RI of cisplatin in the A549/CDDP cells with a reversal multiple of 1.768 (Figure 2(d)). Therefore, the above data indicate that PD‐0332991 can reverse the resistance of the A549/CDDP cells to cisplatin.

### The effect of PD‐0332991 combined with cisplatin on cell cycle distribution and apoptosis

**FIGURE 3:**
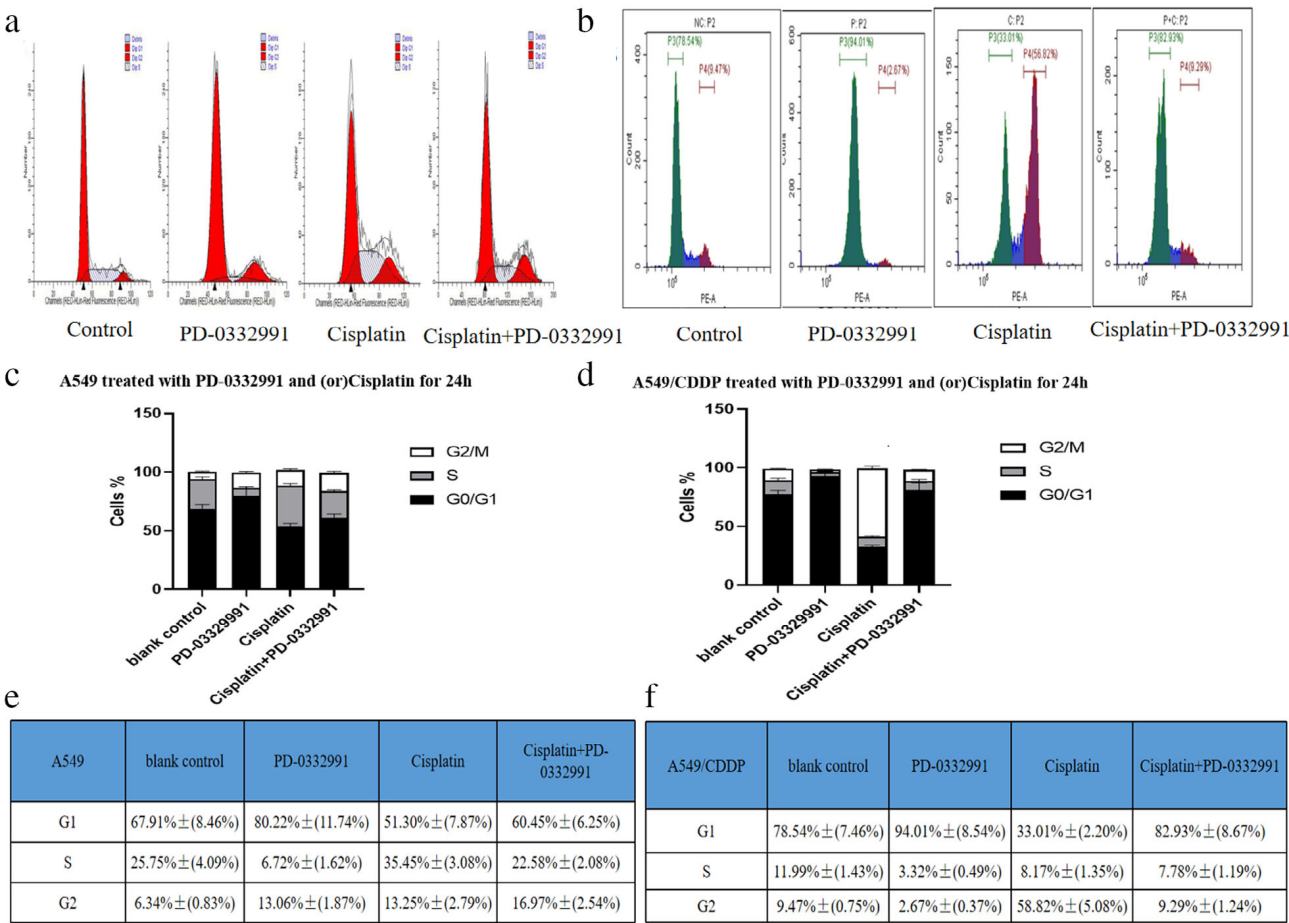
PD‐0332991 enhances cisplatin‐induced G1 phase arrest in the A549 and A549/CDDP cells. (a, c, e) A549 cells were incubated with cisplatin and/or PD‐0332991 for 24 h, stained with PI, and analyzed by flow cytometry. The cell cycle distribution shows the cisplatin + PD‐0332991 group had more G0/G1 phase arrest than the cisplatin group. (b, d, f) the A549/CDDP cells were also incubated with cisplatin and/or PD‐0332991 for 24 h, stained with PI, and analyzed by flow cytometry. The cell cycle distribution shows that the cisplatin + PD‐0332991 group also had more G0/G1 phase arrest than the cisplatin group

First, we employed PI staining to evaluate the cell cycle distribution. As presented in Figure 3(a)–(f), a single treatment of cisplatin led to a 51.3% increase in the G0/G1 arrest in the A549 cells and a 33.01% increase in the A549/CDDP cells. Conversely, the combination of PD‐0332991 and cisplatin caused a 60.45% increase in the G0/G1 arrest in the A549 cells and an 82.93% increase in the A549/CDDP cells. These results indicate that the combination treatment of PD‐0332991 and cisplatin induced a higher rate of G0/ G1 arrest than treatment with cisplatin alone.

**FIGURE 4:**
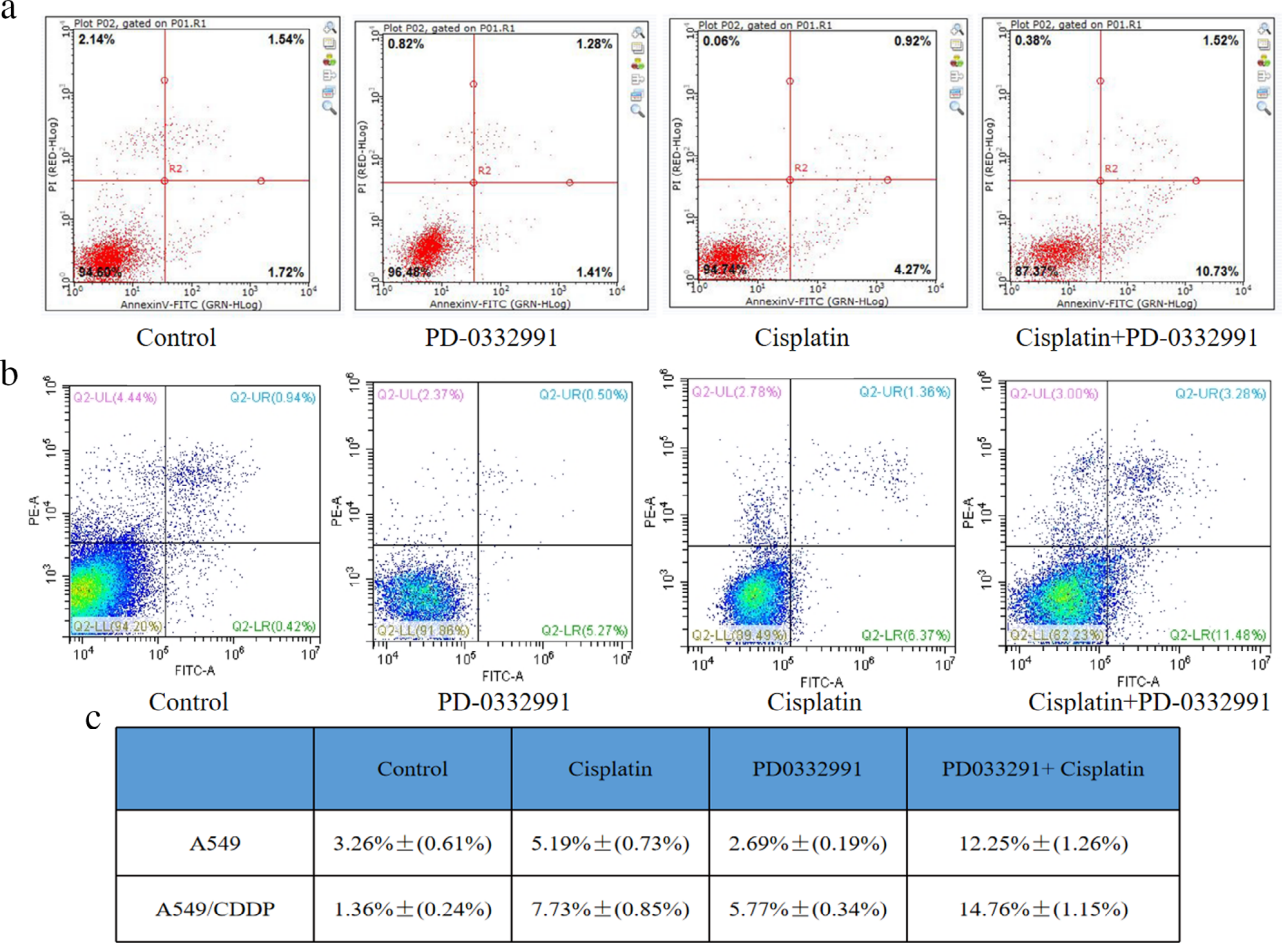
PD‐0332991 enhances cisplatin‐induced apoptosis in cisplatin‐sensitive and cisplatin‐resistant cells. (a, b) After 24 h and after exposure to PD‐0332991 alone, cisplatin alone, or the combination of PD‐0332991 and cisplatin, the A549 and A549/CDDP cells were prepared for AnnexinV/7AAD analysis via flow cytometry. (c) Apoptosis data of the A549 and A549/CDDP cells treated with PD‐0332991 and/or cisplatin for 24 h

Next, we employed AnnexinV/7AAD staining to evaluate cell apoptosis caused by treatment with PD 0332991. As presented in Figure 4(a)–(c), when only cisplatin was used for the A549 and A549/CDDP cells, the apoptosis rates were 5.19% and 7.73%, respectively. However, when the combination treatment of PD‐0332991 and cisplatin was used in the A549 and A549/CDDP cells, the apoptosis rates were 12.25% and 14.76%, respectively. These results indicate that PD‐0332991 can enhance cisplatin‐induced apoptosis.

### The potent mechanism of PD‐0332991 enhances the effects of cisplatin

**FIGURE 5:**
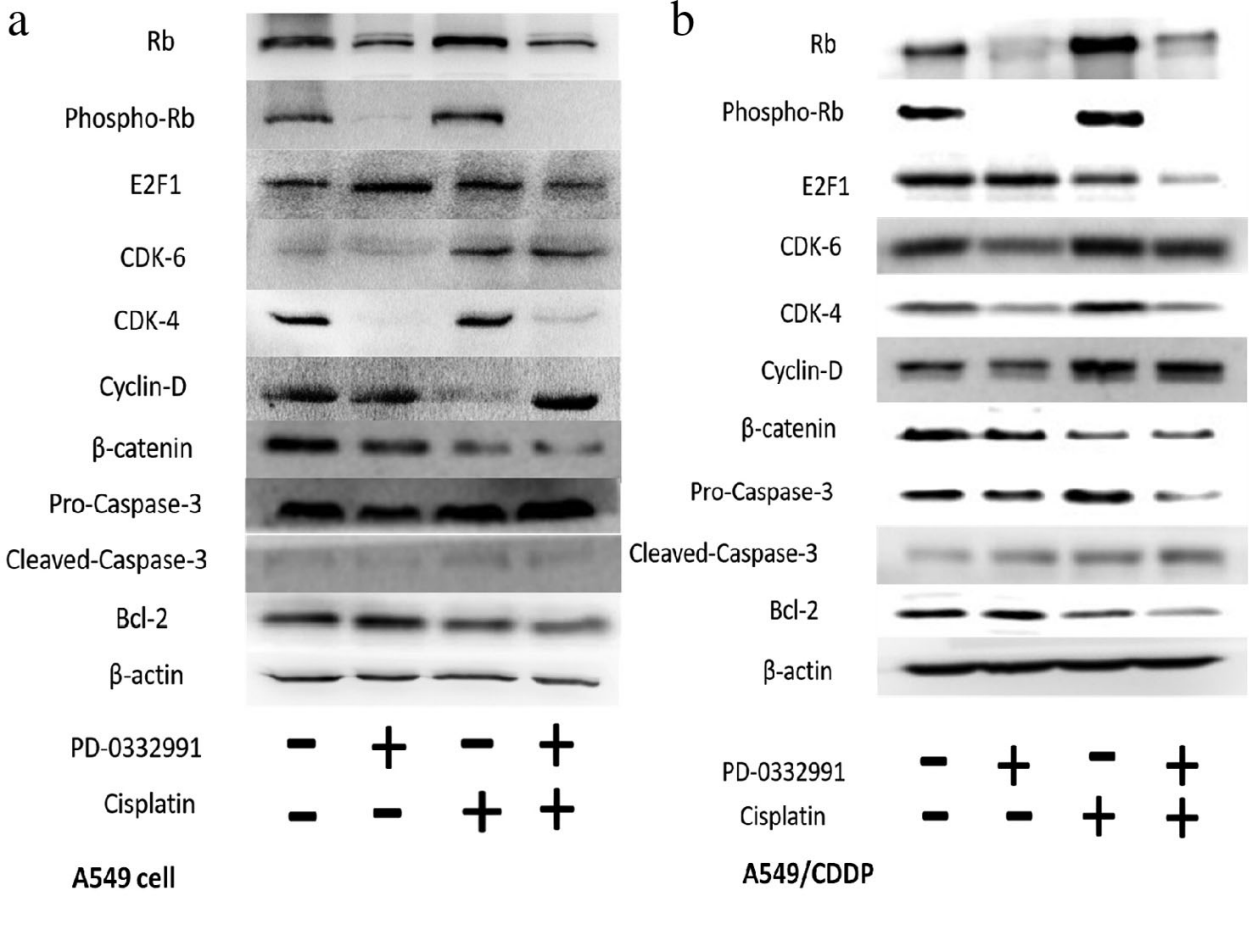
Expression of related proteins after 24 h of treatment in the A549 and A549/CDDP cells. The expressions of Rb, P‐Rb, and E2F1 were lower in the combination group than in the A549 and A549/CDDP cells. However, the protein expression in CDK4/6 and cyclin D was stronger than that in PD‐0332991 alone. Conversely, the expression of the cisplatin resistance‐related protein β‐catenin was inhibited in the combination group in both cells. In the combination group, the expression of pro‐caspase‐3 protein weakened, whereas the expression of cleaved caspase‐3 was enhanced; furthermore, the expression of anti‐apoptotic protein Bcl‐2 was downregulated

To detect the underlying mechanism of the synergistic effect of PD‐0332991 and ciplatin in A549 and A549/CDDP. We employed western blot analysis to determine the relationship between cell cycle proteins and apoptotic proteins, as presented in Figure 5. As demonstrated in the upper panels in Figure 5(a),(b), the expression of cell cycle‐associated proteins Rb, P‐Rb, and E2F1 was expressed the lowest in the combination group in A549 and A549/CDDP compared with control, PD‐0332991, cisplatin groups, but the expression of CDK‐4/6 and Cyclin D was stronger than PD‐0332991 group.

Apoptotic proteins were detected via western blot analysis, as presented in the lower panels in Figure 5(a),(b). In the A549 cell line, the changes in the cleaved caspase‐3 and pro‐caspase‐3 expressions were not significant. However, in the A549/CDDP cells, the expression of the cisplatin resistance‐related protein β‐catenin was inhibited, the expression of pro‐caspase‐3 protein weakened, and the expression of cleaved caspase‐3 improved in the combination group. To further understand the pathways of the anti‐apoptotic effect of PD‐0332991 and cisplatin in NSCLC, we used an anti‐apoptotic protein, namely, Bcl‐2. The results of the western blot analysis revealed that the expression of Bcl‐2 was the lowest in the combination group in cisplatin‐sensitive and cisplatin‐resistant cells compared with the other three groups.

## DISCUSSION

Currently, chemotherapy is the main method employed for the treatment of lung cancer. As a first‐line treatment, platinum‐based chemotherapy can inhibit tumor proliferation and metastasis. However, the drug resistance of lung cancer cells is the major cause of chemotherapy failure and disease progression.^13^ PD‐0332991 is a potent CDK4/6 inhibitor that inhibits the cell cycle arrest of tumor cells in the G1 phase.^14^

In most normal cells, the Rb protein is in a state of low phosphorylation. However, in rapidly proliferating tumor cells, the Rb protein is in a high phosphorylation state. Previous studies demonstrated that the phosphorylation of Rb is related to the G1 to S phase of the cell cycle.^15^ Cell cycle progression is limited by “checkpoints”, G1/S and G2/M. The G1/S checkpoint is primarily regulated by the CDK4/6‐cyclin D and Rb‐E2F complex. When CDK4/6 binds to cyclin D, the complex activates Rb‐E2Fs, catalyzes Rb phosphorylation, and then releases E2F1, which participates in the initiation of gene transcription and promotes the transition from cell cycle G1 to S phase.^16^, ^17^

Furthermore, E2F is a cell cycle‐associated transcription factor, whereas E2F1 is an important transcription factor in cell cycle progression. As a positive regulator of the cell cycle, it can promote the cell cycle from the G1 to the S phase.^18^ E2F1 is also involved in cell apoptosis. Activated E2F1 has the ability to regulate programmed cell death while inducing cell cycle progression. The overexpression of E2F1 can promote tumor cell death.^19^

In the combination group, the expression of the apoptosis‐related protein caspase‐3 significantly increased. Caspase‐3 plays a significant positive regulatory role in apoptosis. Apoptosis‐related protein can cleave poly ADP‐ribose polymerase (PARP), and the enzyme is cleaved into two fragments that bind the zinc finger structure and carboxyl group in the PARP. The phase separation of the catalytic region leads to the inactivation of the polymerase. However, it can increase endonuclease activity and cleave DNA, which results in apoptosis.^20^ Bcl‐2 is an anti‐apoptotic protein present in the endoplasmic reticulum of the cells. Its overexpression can inhibit the release of Ca2^+^ from the endoplasmic reticulum, and the release of Ca2^+^ is the initiator in apoptosis.^21^ Bcl‐2 can also protect the plasma membrane by inhibiting oxygen‐free radical production through antioxidant activity.^22^

Studies have demonstrated that the Wnt/β‐catenin signaling pathway plays a significant role in tumor chemotherapy resistance. The upregulation of the Wnt/β‐catenin signaling pathway is associated with cisplatin resistance.^23^ When the Wnt signaling pathway is activated, it can bind to the frizzled receptor, inhibit GSK‐3β phosphorylation, and increase the expression of β‐catenin in the cytoplasm. Our western blot analysis revealed that the combined group β‐catenin exhibited the lowest expression among all four groups, indicating that the cell had become sensitive to cisplatin.

In conclusion, our previous studies demonstrated that PD‐0332991 could reverse EGFR‐TKI‐acquired resistance via downregulation of proliferation, apoptosis induction, and G0/G1 cell cycle arrest. In the clinical setting, cisplatin resistance affects the survival duration of lung cancer patients. We found that the function of cisplatin is associated with the cell cycle. We also attempted to use a CDK4/6 inhibitor, PD‐0332991, combined with cisplatin to reverse cisplatin resistance. Our research revealed that PD‐0332991 could reverse cisplatin resistance using CCK‐8 and the CalcuSyn2.0 software. PD‐0332991 combined with cisplatin can cause cell apoptosis and cell cycle arrest in the G1 phase. Western blot analysis verified the regulation with the cyclinD1/CDK4/6‐Rb/E2F1 pathway. The results are similar to the findings of our previous study.

## AUTHOR CONTRIBUTIONS

M.L., H.L., and J.C. wrote the manuscript. M.L., L.C., X.L., C.X., Y.L., F.R., and R.W. performed the experiments and collected the data. All authors analyzed the data, discussed the data, and revised the article. All authors read and approved the final manuscript.

## CONFLICT OF INTEREST

The authors declare no conflicts of interest.
